# Identification and analysis of insulin like peptides in nematode secretomes provide targets for parasite control

**DOI:** 10.6026/97320630012412

**Published:** 2016-12-14

**Authors:** Shachi Gahoi, Budhayash Gautam

**Affiliations:** 1 Department of Computational Biology and Bioinformatics, Sam Higginbottom Institute of Agriculture Technology & Sciences,Allahabad, 211007, India

**Keywords:** Insulin like proteins, Neuropeptides, Parasite control, Genomics, Drug targets

## Abstract

Insulin-like (ins) peptides play an important role in development and metabolism across the metazoa. In nematodes, these are also required for dauer formation and longevity and are expressed in different types of neurons across various life stages which demonstrate their role in parasites and could become possible targets for parasite control. To date, many nematode genomes are publically available. However, a systematic screening of ins peptides across different nematode group has not been reported. In the present study, we systematically identified ins peptides in the secretomes of 73 nematodes with fully sequenced genomes covering five different groups viz. plant parasitic, animal parasitic, human parasitic, entomopathogenic and free living nematodes. From the total of 93,949 secretory proteins, 176 proteins were uniquely mapped to 40 identified C. elegans ins families. The obtained result showed that 74.15% of the identified ins proteins were represented in free living nematodes only and remaining 25.84% were combinedly identified in all other nematode groups. The ins-1, ins-17 and ins-18 were the only ins families which were detected in all the studied nematode groups. Out of 176 proteins, 96 of ins proteins were predicted as hydrophilic in nature and 39 proteins were found stable using ProtParam analysis. Our study provides insight into the distribution of ins peptides across different group of nematodes and this information could be useful for further experimental study.

## Background

Neuropeptides are short sequences of amino acids that are released from nerve cells and function in multicellular organisms to communicate information between cells. In nematodes, these neuropeptides fall into three distinct groups, the FMRFamide-like peptide family encoded on flp genes, the insulin-like peptide family encoded on ins genes and all other neuropeptide-like proteins encoded on nlp genes. Insulin like peptides (INSs) play an important role in daur formation, metabolism and other development process. These were also involved in limiting body size and determining lifespan [1,2].

Ins genes are expressed in different types of neurons across numerous stages of life - embryos, larvae and adult worms. To date, 40 genes encoding insulin-like peptides have been identified in C. elegans. DAF-2, an insulin-like peptide receptor mediates a pathway determining dauer formation [3]. Ins-1, ins-9 and daf-28 are expressed in ASI and ASJ chemosensory neurons which are critical for the decision of dauer formation, as well as other neurons. Ins-1 and ins-9 loss of function mutants reported no dauer phenotype while over expression caused a low level of dauer arrest in wildtype animals which indicate that ins-1 and ins-9 peptides can modulate the DAF-2 signaling pathway and stimulate dauer formation [4]. Involvement of ins peptides in dauer formation, development processes and aging regulation in C. elegans demonstrate their role in parasites and potential of their signaling pathways as possible drug targets. Interestingly, in planta-based RNAi control strategies could be used for parasite control whereby neuropeptide encoding genes could become possible targets.

The prediction of insulin like peptides from sequenced genome could be used to prioritize the experimental analysis of potential targets of parasite control. The increasing availability of whole genome of nematode organisms provides the opportunity to systematically screen their secretome for ins genes using bioinformatics approaches. To date, various insulin-like peptides have been reported in different nematodes. However, a systematic genome-wide identification and comparative analysis of ins peptides across the nematodes is required to understand their distribution in different groups of nematodes. In this study, we have identified and compared the full repertoires of ins peptides in secretomes of 73 nematodes belonging to different groups. In addition, we detected over-represented motifs in all the identified ins peptides and also performed physico-chemical properties analysis. Our study is the most comprehensive in silico collection of nematodes ins peptides and provides valuable resource for further experimental studies for parasite control.

## Methodology

### Data collection and identification of insulin like proteins in nematode secretome

The forty C. elegans ins proteins were identified and downloaded from the wormbase database [5]. As ins proteins contain a signal peptide sequence, we used in-house data of 93,949 secretory proteins, identified from 73 nematodes (unpublished data). In order to search ins proteins in nematode genomes, 93,949 secretory proteins were compared with forty c. elegans ins proteins using BLASTP algorithm [6] at an E-value cut-off of 1e- 05. Cd-hit [7] was used to remove redundant proteins at 100 % similarity.

### Identification of over-represented motifs, gene ontology and functional domains

We discovered over represented motif consensus pattern in all the identified ins proteins using the Multiple Expectation maximization for Motif Elicitation analysis tool (MEME v.4.11.2) [8]. This program was used to search best 5 motif consensus patterns of 6–50 bases width. The gene ontology and functional domains of the predicted ins proteins were identified using standalone version of InterProScan 5 (v.5.17-56.0) with default parameters [9].

### Multiple sequence alignment and Physico-chemical properties analysis

Multiple alignment was performed with the muscle algorithm, using standard parameter and the phylogenetic relation among the obtained aligned sequences were analyzed based on neighbor joining method using MEGA 6 [10]. The physico-chemical properties such as molecular weight, amino acid composition, instability index, isoelectric point, aromaticity, grand average of hydropathicity (GRAVY) of the identified ins proteins were calculated by using ProtParam software [11].

## Discussion

### Whole secretome identification and distribution of ins proteins

**Figure 1:**
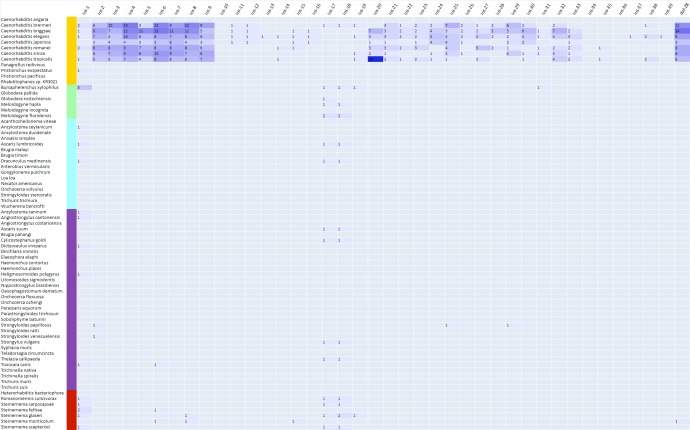
Distribution of identified ins proteins in different group of nematodes. Yellow, green, cyan, purple and red color represent free living, plant parasitic, human parasitic, animal parasitic and entomopathogenic nematodes respectively. The result showed overrepresentation of the identified ins proteins in free living nematodes. Family ins-1, ins-17 and ins-18 were detected in all the studied nematode groups.

To gain insight into the distribution of ins proteins in nematodes, the secretomes of 73 nematode from various groups were systematically screened. These nematodes represent five different types of group, plant parasitic nematode, animal parasitic nematode, human parasitic nematode, entomopathogenic nematode and free living nematode. From the total of 93,949 secretory proteins, 788 proteins were mapped to C. elegans ins proteins at an E-value cutoff of 1e-05 (Figure 1). These 788 proteins contain redundancy because one protein could map to different ins proteins. Removing duplicates by cd-hit resulted in 176 unique ins proteins which were used for further analysis.

The distribution of identified 176 ins proteins in plant parasitic, animal parasitic, human parasitic, EPN and free living nematodes were 11, 11, 5, 19 and 130 respectively. The obtained result showed that 74.15% of the identified ins proteins were represented in free living nematodes only and remaining 25.84% were combinedly identified in all other nematode groups. The ins-1, ins-17 and ins-18 were the only ins families which were detected in all the studied nematode groups while ins-4, ins-34 and ins-36 families were not identified in any studied nematodes.

### Physico-chemical properties analysis

The physico-chemical properties were calculated for the complete set of identified ins sequences (Table 1 – available with authors). The predicted isoelectric point revealed INS-1 and INS-17 proteins to be more basic in nature compared to other identified INS proteins. The calculated values of isoelectric point value will be useful for separating the INS protein on a polyacrylamide gel by isoelectric focusing. The stability of identified INS proteins was studied by analyzing the instability index value. The minimum, maximam and average value of instability index for identified INS proteins were 18.55, 83.37, 51.41. Out of 176 proteins, 39 proteins were found stable and remaining proteins were predicted as unstable. The solubility of proteins was analysed by the grand average of hydropathicity (GRAVY) index. The negative GRAVY value designates proteins to be hydrophilic in nature, hence 96 of INS proteins were predicted as hydrophilic.

### Functional domain, gene ontology and conserved motif analysis

The InterproScan analysis detected insulin-like domain (IPR016179) in all the identified INS proteins. In addition, INS proteins were functionally categorized into hormone activity (GO:0005179) of molecular function and extracellular region (GO:0005576) of cellular component category with gene ontology terms. The MEME analysis predicted over-represented motifs in each sequence of predicted INS proteins. The details for all the identified motifs, motif regular expression, log likelihood ratio (llr), E-value, motif conservation and motif logo are given in Table 2 (available with authors).

### Expansion of the INS-1 family in nematode secretomes

**Figure 2:**
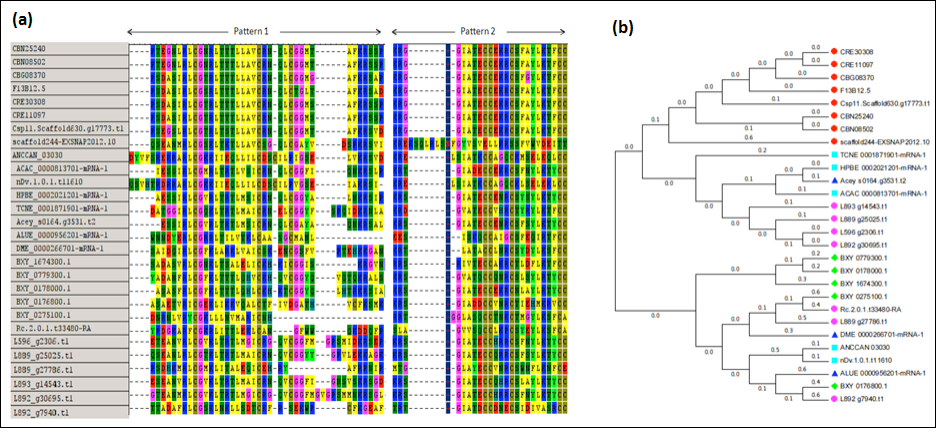
Sequence and phylogeny analysis of identified ins-1 family members (a) Multiple sequence alignment of ins-1 family members showed conserved signature patterns. (b) Phylogenetic tree based on alignment of ins-1 family members. Red, cyan, blue, green and pink nodes represent free living, animal parasitic, human parasitic, plant parasitic and entomopathogenic nematodes respectively. Members of free living nematodes were grouped into one cluster. Few of the members from animal parasitic, human parasitic and entomopathogenic nematodes were grouped into second cluster. Plant parasitic members and remaining members from animal parasitic, human parasitic and entomopathogenic nematodes were grouped into third cluster.

Of the 93,949 secretory proteins, 28 proteins were mapped to C. elegans INS-1 proteins. These 28 proteins were found to be distributed in 20 studied nematodes belonging to all the five nematode groups, plant parasitic, human parasitic, animal parasitic, EPN and free living nematodes. Multiple sequence alignment of all these members of ins-1 proteins has been performed. The obtained alignment showed conserved signature patterns of ins-1 protein with slight sequence variation (Figure 2a). Phylogenetic analysis showed that all the members of ins-1 family were grouped into three clusters. Members of free living nematodes were grouped into one cluster. Few of the members from animal parasitic, human parasitic and entomopathogenic nematodes were grouped into second cluster while plant parasitic members and remaining members from animal parasitic, human parasitic and entomopathogenic nematodes were grouped into third cluster (Figure 2b).

**Figure 3:**
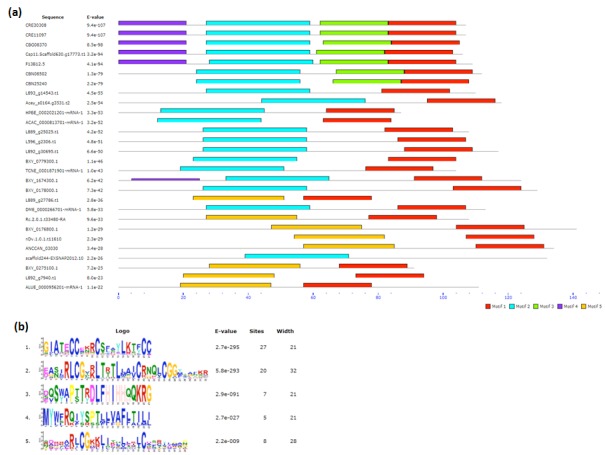
Conserved motif analysis using MEME. (a) Conserved motifs found in identified 27 members of ins-1 family. (b) Motif logos for identified conserved motifs.

The MEME analysis was used to find over-represented motifs in complete set of INS-1 proteins (Figure 3a). Among the top five motifs identified by this analysis, first motif was shared by 27 members and second motif was shared among 20 members of ins-1 proteins. These two motifs represent conserved signature pattern of ins-1 proteins (Figure 3b). Futhermore, third and fourth motifs were found to be specific in free living nematodes while the fifth motif was distributed in eight members from all the studied nematode group except free living nematodes.

## Conclusion

The present study provides secretome-wide identification, functional annotation and conserved motif analysis of insulin like proteins in 73 nematodes. In silico analysis revealed that of the 93,949 secretory proteins, 176 proteins were mapped to forty C. elegans insulin like proteins. The insulin-like domain (IPR016179) was detected in all the identified ins proteins which strengthen our computational identification approach. Ins-1, ins-17 and ins- 18 families were identified in all the studied nematode groups while ins-4, ins-34 and ins-36 families were found to be absent in any studied nematodes. The MEME analysis showed conserved signature patterns in all the identified members of ins-1 family. The isoelectric point predicted using ProtaParam software revealed INS-1 and INS-17 proteins to be more basic in nature compared to other identified INS proteins. Our study is the most comprehensive computational analysis of the insulin like peptides in nematode genomes and could be useful for further experimental study.
